# Discovery of Unusual Cyanobacterial Tryptophan-Containing Anabaenopeptins by MS/MS-Based Molecular Networking

**DOI:** 10.3390/molecules25173786

**Published:** 2020-08-20

**Authors:** Subhasish Saha, Germana Esposito, Petra Urajová, Jan Mareš, Daniela Ewe, Alessia Caso, Markéta Macho, Kateřina Delawská, Andreja Kust, Pavel Hrouzek, Josef Juráň, Valeria Costantino, Kumar Saurav

**Affiliations:** 1 Laboratory of Algal Biotechnology-Centre Algatech, Institute of Microbiology of the Czech Academy of Sciences, 37981 Třeboň, Czech Republic; saha@alga.cz (S.S.); urajova@alga.cz (P.U.); mares@alga.cz (J.M.); ewe@alga.cz (D.E.); macho@alga.cz (M.M.); delawska@alga.cz (K.D.); kust@alga.cz (A.K.); hrouzek@alga.cz (P.H.); 2 Task Force Big Fed2, The Blue Chemistry lab, Università degli Studi di Napoli, 80131 Napoli, Italy; germana.esposito@unina.it (G.E.); alessia.caso@unina.it (A.C.); 3 Institute of Hydrobiology, Biology Centre of the Czech Academy of Sciences, 37005 České Budějovice, Czech Republic; 4 Faculty of Science, University of South Bohemia in České Budějovice, Branišovská 1760, 37005 České Budějovice, Czech Republic; josef.juran@ibot.cas.cz; 5 Institute of Botany of the Czech Academy of Sciences, 252 43 Průhonice, Czech Republic

**Keywords:** *Brasilonema*, anabaenopeptins, hexapeptides, tryptophan-containing peptides, molecular networking, antiproliferative activity

## Abstract

Heterocytous cyanobacteria are among the most prolific sources of bioactive secondary metabolites, including anabaenopeptins (APTs). A terrestrial filamentous *Brasilonema* sp. CT11 collected in Costa Rica bamboo forest as a black mat, was studied using a multidisciplinary approach: genome mining and HPLC-HRMS/MS coupled with bioinformatic analyses. Herein, we report the nearly complete genome consisting of 8.79 Mbp with a GC content of 42.4%. Moreover, we report on three novel tryptophan-containing APTs; anabaenopeptin 788 (**1**), anabaenopeptin 802 (**2**), and anabaenopeptin 816 (**3**). Furthermore, the structure of two homologues, i.e., anabaenopeptin 802 (**2a**) and anabaenopeptin 802 (**2b**), was determined by spectroscopic analysis (NMR and MS). Both compounds were shown to exert weak to moderate antiproliferative activity against HeLa cell lines. This study also provides the unique and diverse potential of biosynthetic gene clusters and an assessment of the predicted chemical space yet to be discovered from this genus.

## 1. Introduction

For 3.5 billion years, cyanobacteria have kept developing new adaptation features and defense mechanisms which allowed them to colonize the earth, shape its atmosphere from anoxic to oxygenic, and ever since to survive in harsh and strongly competitive environments under extreme temperatures, salt stress, high UV-radiation, and pathogen attack [1,2]. One of these survival strategies is the production of a vast variety of secondary metabolites, exhibiting a broad spectrum of biological activities and properties, including peptides, lipopeptides, polyketides, alkaloids, lipids, and terpenoids [3,4,5,6,7]. When growth conditions are advantageous, cyanobacteria proliferate, resulting in overgrown populations known as cyanobacterial blooms (CBs), which can be harmful for aquatic life as well as for human health because of the toxins they produce [8,9]. Cyanobacteria comprise a wide range of phylogenetic lineages, of which the monophyletic order Nostocales (heterocytous cyanobacteria) are an extremely competent group in terms of general secondary metabolite production [10]. Filamentous cyanobacteria, in particular the genera *Anabaena*, *Nostoc*, *Oscillatoria*, and *Lyngbya*, produce a large variety of secondary metabolites, estimated to account for approximately 25% of the total number of known cyanobacterial metabolites [11,12,13,14]. These compounds are usually produced by nonribosomal peptide synthetases (NRPS), polyketide synthases (PKS), or NRPS/PKS hybrid enzymes. Recent advancement in the field of genome sequencing and the rise of fast and extremely accurate analytical methods, such as high-performance liquid chromatography connected to high-resolution mass spectrometry (HPLC-HRMS/MS), have accelerated the discovery of novel secondary metabolites e.g., [5,6,15]. The use of molecular networking as a dereplication strategy allows to identify the complexity of variants produced by respective biosynthetic genes, which in turn produce valuable data for pharmaceutical development, since structural variants often have different biological activities [16,17,18]. 

Anabaenopeptins (APTs) are a family of cyclic hexapeptides produced by diverse cyanobacterial taxa, first described from the planktonic representative *Anabaena flos-aqae* NRC 525-17 [19]. APTs manifest a general structure X^1^-CO-[Lys^2^-X^3^-X^4^-MeX^5^-X^6^], wherein the X1 and X3-6 can be either non-proteinogenic or proteinogenic amino acids. The cyclization bond is formed between the *C*-terminal carboxyl and the *N*-terminal amine of the conserved D-Lys at position 2 [20]. The exocyclic amino-acid X1 is connected to the α-amino group of the conserved Lys residue via the ureido bond. APTs encoded in the biosynthetic gene cluster first identified from *Planktothrix rubescens* NIVA CYA 98 [21] are produced by NRPS following a sequential synthesis pathway using (non) proteinogenic amino acids as a substrate [22]. These clusters have been characterized in many strains belonging to various genera such as *Anabaena*, *Nostoc*, *Nodularia,* and *Planktothrix* [21,22,23,24]. Large structural variability is observed among APTs with members exhibiting masses between 679 and 956 Da, with various biological activities, including inhibition of protein phosphatase, carboxypeptidases A and U, and other protease-inhibitory activity [20]. Since the first study, more than 115 isoforms of APTs have been reported, out of which 104 are produced by cyanobacteria, belonging to the genera *Anabaena*, *Aphanizomenon*, *Microcystis*, *Nodularia*, *Nostoc*, *Planktothrix, Oscillatoria, Schizothrix* and *Lyngbya* [25].

In the present study, we report the draft genome sequence of a new *Brasilonema* strain collected in Costa Rica. Furthermore, the combined approach of genome mining and HPLC-HRMS/MS coupled with bioinformatic analyses led us to identify four unusual novel tryptophan-containing APTs showing moderate antiproliferative activity. 

## 2. Results and Discussion

**Figure 1:**
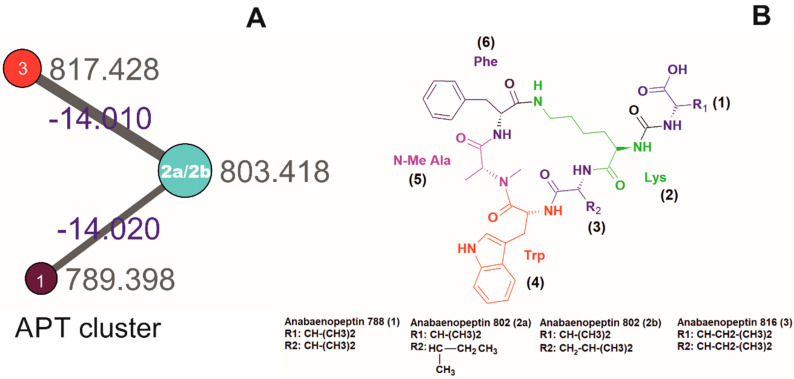
(**A**). The molecular network with anabaenopeptins (APT) cluster obtained from the organic extract of *Brasilonema* sp. CT11. Nodes are labeled with parent *m*/*z* values and edges with the mass difference. Node size is indicative of the ion count; edge thickness is relative to cosine score. (**B**). Four APTs detected using LC-HRMS/MS following general structure X^1^-CO-[Lys^2^-X^3^-X^4^-MeX^5^-X^6^], wherein the X1 and X3-6 are non-proteinogenic or proteinogenic amino acids.

Strains belonging to the genus *Brasilonema* are a good source of diverse secondary metabolites [26]. The genus *Brasilonema*, belonging to the family Scytonemataceae, are terrestrial filamentous, heterocytous false-branching cyanobacteria previously isolated from subtropical and tropical climate zones [27,28,29]. To the best of our knowledge, the only tryptophan-containing APTs were detected in 2015 from the genus *Brasilonema* sp. (CENA360 and CENA382) isolated from Brazilian Atlantic Coastal Forest [30], and a hydroxyl-tryptophan containing APT mozamide A, isolated from Theonellid sponge from Mozambique [31]. In this study, we analyzed a terrestrial filamentous *Brasilonema* sp. CT11 collected in Costa Rica, as black mat on a bamboo trunk (Figure S1). The crude extract (~16 mg) was obtained upon one week of cultivation in 300 mL glass column under continuous light condition and its subsequent solvent extraction. The molecular network formed by GNPS analyses yielded thirty clusters and one hundred and eight single nodes without grouping (Figure S2). To date, >3000 million MS/MS spectra from various instruments (e.g., Orbitrap and qTOF) have been searched at GNPS, yielding putative dereplication matches of 7.7 million spectra to more than 35,477 compounds. A schizopeptins-related cluster was present in the network and is shown in Figure 1, forming a cluster representing three nodes with *m*/*z* 817.428, *m*/*z* 803.418, and *m*/*z* 789.398. Furthermore, the manual curation and detailed MS/MS spectra study led us to interpret the putative presence of three novel tryptophane-containing APTs: anabaenopeptin 788 (*m*/*z* 789.4425 [M + H]^+^), anabaenopeptin 802 (*m*/*z* 803.4417 [M + H]^+^), and anabaenopeptin 816 (*m*/*z* 817.4425 [M + H]^+^). The structures of these compounds were manually curated and postulated in accordance with the fragment ions listed in Table 1.

As a complementary approach to GNPS, we applied whole genome sequencing and subsequent genome mining to investigate the genetic potential of *Brasilonema sp*. CT11 for the production of secondary metabolites. The newly sequenced draft genome of *Brasilonema* CT11 (NCBI accession number JABXYX010000000) was retrieved in 411 genomic scaffolds of approximately 8.79 Mbp of total length (99.48% estimated completeness) and 42.4% average GC content. The assembly was analyzed for the presence of secondary metabolite biosynthetic gene clusters (BGCs) using antiSMASH 5.1.1. AntiSMASH uses Hidden Markov Models (HMM) and rules-based detection to identify a broad array of BGCs, including those encoding polyketide, non-ribosomal peptides, terpenes, aminoglycosides and ribosomally synthesized and post-translationally modified peptides (RiPPs) from bacterial genomes. AntiSMASH predicted 36 putative secondary metabolite clusters from *Brasilonema* CT11. Out of the 36 gene clusters, four clusters possess 100% similarity with other known BGCs whereas only two clusters were detected with more than 75% similarity with known BGCs (Table S1) [32]. Most of these gene clusters were in the NRPS category with 12 recovered BGCs. These putative BGCs yielded hits among various known secondary metabolites showing variable similarity scores, ranging from maximum similarity with known natural product Anabaenopeptin 908/915 (100%) [22], to minimum similarity with nostocyclopeptide A2 (28%) [33], suggesting that the pathways may encode new natural products or natural products with no characterized BGCs. Next to NRPS the second most abundant BGCs were NRPS-PKS hybrid (6 clusters), of which one cluster showed 100% similarity to nostopeptolide A2 [34]. Another interesting hybrid BGC with 60% similarity to hapalosin BGC [35] was detected, which may lead to the discovery of a new cyclic peptide. Besides these, the other major BGCs detected were: one cluster of PKS type I, three terpenes, two RiPPs, four bacteriocins, and one indole. Among RiPPs class, one lassopeptide and one lantipeptide were detected with no similarity with any reported gene clusters. The PKS type I gene cluster, Merocyclophane C/D [36] was detected with highest similarity of 22%, which is particularly interesting precisely because of its low similarity. Production of a vast arsenal of secondary metabolites detected in *Brasilonemma* CT11 is in agreement with the large variety of peptides reported in other *Brasilonemma* strains as well as related branched cyanobacteria of genus *Scytonema* [20]. However, to the best of our knowledge no data on secondary metabolites production are available for related genera *Symphonemopsis* and *Symphonema* which makes the comparison impossible.

**Figure 2:**
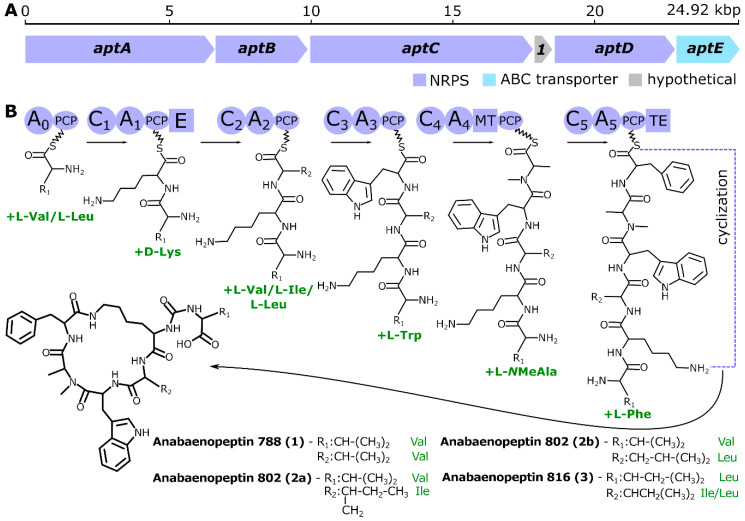
Anabaenopeptin synthetase gene cluster arrangement of *Brasilonema* CT11 yielding four anabaenopeptins **1**, **2a**, **2b,** and **3**. (**A**) Gene map of the apt biosynthetic gene cluster. (**B**) Proposed biosynthesis of anabaenopeptins in *Brasilonema* sp. CT11. A—adenylation domain; C—condensation domain; E—epimerization domain; MT—methyltransferase domain; PCP—peptidyl-carrier protein; TE—thioesterase domain.

**Figure 3:**
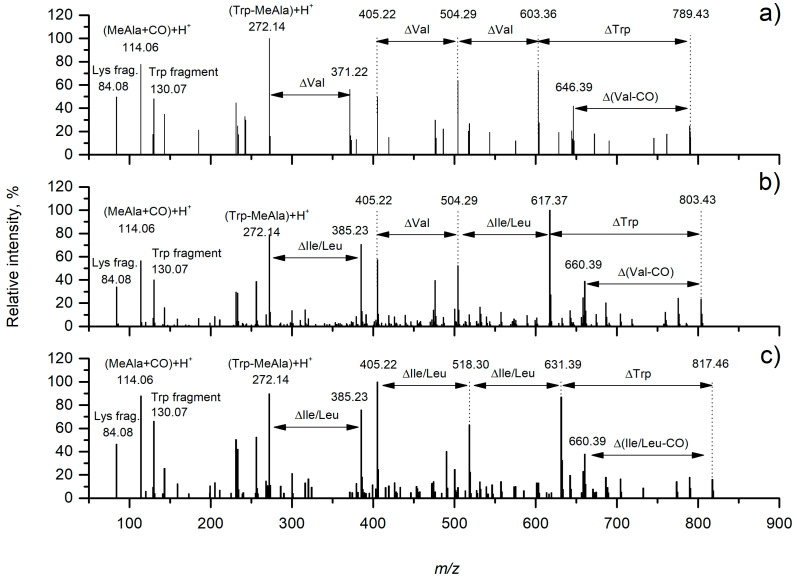
HR-MS/MS product ion spectra of protonated anabaenopeptins from *Brasilonema* CT11; (**a**) compound **1**, (**b**) compound **2a/2b**, and (**c**) compound **3**.

The discovery of a complete putative APT BGC (NCBI accession number MT670293) along with the detection of unusual Trp-containing APT variants using MS/MS networking coupled with manual curation encouraged us to investigate this gene cluster in detail. The putative APT BGC was nearly 25 kbp long and consisted of five genes (*aptA–E*), clearly homologous to previously reported *apt* genes (Table S2., [23,25]), and a single short hypothetical open reading frame (Figure 2). Adenylation domains (A-domains) present in the six encoded NRPS modules (Table S3) showed predicted substrate specificities that were in agreement with the amino-acid residues observed in the APT variants detected herein (Figure 3, Table 1), including an unequivocally predicted Trp residue at position 4 of the peptide cycle. Intriguingly, in silico substrate prediction for the starter A-domain (A_0_) and the third A-domain (A_2_) reported only a broad specificity to hydrophobic aliphatic amino acids, with Val, Leu, and Ile among the most probable candidates based on algorithms implemented in antiSMASH. These results are in line with the observed substrate promiscuity, yielding APT variants having either Val or Leu at position 1 and Ile, Leu, or Val at position 3. Previous reports have indicated both substrate promiscuity and occurrence of alternative starter modules to be responsible for variability in amino acids incorporated into APTs [22,23], however we have not detected any additional alternative starter module in our genomic scaffold. The genomic data suggest d-Lys based on the presence of an epimerase (E) domain in the corresponding biosynthetic NRPS module, the remaining amino acids are predicted to be l-enantiomers (Figure 2).

Furthermore, fractionation and manual curation of the spectra suggested to us the presence of four unusual tryptophan-containing anabaenopeptins (two homologues with *m*/*z* 803.4417 [M + H]^+^, and 803.4425 [M + H]^+^, one having *m*/*z* 789.4425 [M + H]^+^ and one with *m*/*z* 817.4425 [M + H]^+^) using LC-HRMS/MS. The structures of these compounds were postulated in accordance with the fragment ions listed in Table 1 and product ion spectra depicted in Figure 3. Subsequent chromatographical separation led us to isolate two homologues with *m*/*z* 803.44 [M + H]^+^, **2a** (0.6 mg) and **2b** (2.6 mg). 

**Figure 4:**
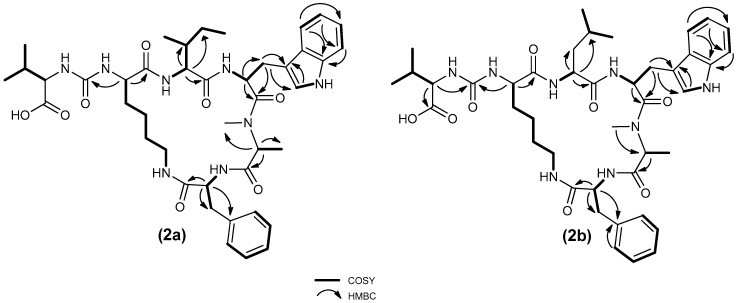
Most significant correlations provided by COSY and HMBC 2D spectra of compounds **2a** and **2b**.

The HR-ESI-MS mass spectrum of compounds **2a** and **2b** showed [M + H]^+^ pseudomolecular ion peaks at *m*/*z* 803.4417 and 803.4425 respectively, which defined its molecular formula as C_42_H_58_N_8_O_8_. Their MS/MS spectra were identical. The fragmentation pattern was indicative of two cyclic peptide compounds, with fragments *m*/*z* 504.28 which indicated the presence of Val at position 1, Lys at position 2, MeAla at position 5, and Phe at position 6; and *m*/*z* 385. 22 which indicated the presence of Ile/Leu at position 3, and Trp at position 4. Binding of leucine or isoleucine cannot be distinguished by conventional mass spectrometry HPLC-purification was therefore performed to obtain two compounds in pure state. The structures of both homologues were confirmed by extensive NMR analysis. A full set of homonuclear and heteronuclear two-dimensional NMR spectra (COSY, NOESY, HSQC, and HMBC) was recorded (Tables S4 and S5, Figures S4–S15). In particular, the proton spectra showed seven amide NH signals and six different α-proton signals, as expected for a hexapeptide with a *N*-methyl alanine, a lysine, and a tryptophan residue. Analysis of the TOCSY spectrum (Figure S9 and S15) showed correlation of all the protons of the six side chains with the corresponding α-proton. This information combined with that derived from the analysis of COSY, HMBC, and NOESY spectra confirmed the structures of compounds **2a** and **2b**, and the analysis of COSY spectra led to distinguish the presence of an isoleucine and a leucine residue in compound **2a** and **2b** respectively (Figure 4). 

Taken together, MS evidence, ^1^H-NMR and 2D-NMR data, allowed the identification of compounds **2a** and **2b**, designated as anabaenopeptin 802a and anabaenopeptin 802 b, respectively (Figure 4). The elucidated structure indicated that compounds **2a** and **2b** are structurally identical to the compounds reported previously [30]. However, the complete structural elucidation using NMR spectroscopy and its biological activity determination is reported now for the first time.

**Figure 5:**
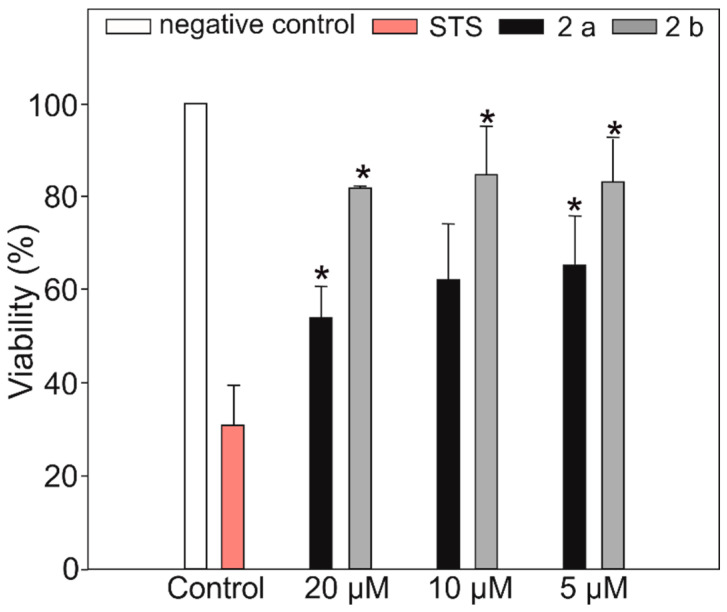
Cell viability was assessed by MTT assay at 48 h exposure time. Cell viability was calculated as the percentage of viable cells in **2a** and **2b** compounds-treated cells relative to control at 20, 10, and 5 µM concentration. Data are presented as mean ± SD; *n* = 3. * *p* ≤ 0.05. STS stands for Staurosporine, used as positive control at 1 µM concentration.

The viability assay was performed on HeLa cells after 48 h of exposure time (Figure 5). The results show differences between the effectiveness of two structural variants **2a** and **2b**. **2a** exhibited a slightly stronger cytostatic effect at concentrations 20, 10 and 5 µM than **2b**. There was no significant difference observed between the effectivity of both the compounds at lower concentrations (2.5, 1.25 and 0.625 µM) with no decrease in viability (data not shown). These results suggest that even the small conformational change due to the occurrence of alternative starter modules in APTs BGC can influence the effectiveness of the compounds against cancer cells. Substitutions of the amino acid residues are thought to determine the degree of bioactivity [37,38]. However, more detailed studies on Trp-containing APTs are needed to establish the structure activity relationship. 

## 3. Materials and Methods 

### 3.1. Cyanobacterial Strain and Culturing Conditions 

The *Brasilonema* strain was collected in Costa Rica, as black mat on a bamboo trunk, on March 18, 2010 by Jan Mareš and isolated into culture by Josef Juráň. Strain was grown in Z’ medium in glass columns (300 mL) bubbled with air enriched in 1.5% CO_2_ at constant temperature and illumination of 28 °C and 50 μmol photon m^−2^ s^−1^ [5]. Biomass was harvested by centrifugation (3125× *g*), stored at −80 °C, and subsequently lyophilized.

### 3.2. Genome Sequencing, Assembling, Annotation, and Mining for Identification of Anabaenopeptin Gene Cluster

Single filaments of *Brasilonema* sp. CT11 were isolated by glass capillary technique described previously [39]. Briefly, filaments were serially washed in ten drops of TE buffer and amplified by multiple-displacement amplification (MDA) using the Repli-G Mini Kit (Qiagen). Ten filaments that passed quality check by 16S rRNA sequencing, were pooled together and sent for commercial de novo genome sequencing (EMBL Genomics Core Facility, Heidelberg, Germany) using an Illumina MiSeq Pair-End library with 250 bp reads, 350 bp average insert length, and 1.4 Gbp data yield. The data from Illumina were assembled using SPAdes 3.14 de novo assembler [40] with the single cell option enabled protein coding genes were predicted using Prodigal in the assembled scaffolds longer than 999 bp. All predicted proteins were compared to the NCBI-nr database using MMSeqs26 and only contigs that had the most hits to the cyanobacteria were kept for the rest of the analysis. Completeness of the cyanobacterial bin was estimated using CheckM [41]. The anabaenopeptin gene cluster was identified based on BLASTp searches against the *Brasilonema* sp. CT11 genome assembly, using previously published *apt* genes as queries. Predicted open reading frames in the target genomic scaffold were translated and subjected to BLASTp, NCBI conserved-domain search and antiSMASH 5.0 [32] analysis for functional annotation. 

### 3.3. Crude Extract Preparation

Lyophilized biomass (~100 mg) was grinded (with sea sand) and extracted with 10 mL of following solvents; hexane, chloroform, chloroform: methanol (1:1) and 70% methanol in water. All the extractions with different solvents were done in separate batches. Extracts were sonicated for 10 min (ultrasonication bath) and filtered through glass microfiber filter (1.2 µm). All filtrates containing organic phase solvents were evaporated under vacuum using a rotary vacuum evaporator (Laborota 4002, Heidolph, Germany) 

### 3.4. HPLC-HRMS/MS Analysis

Crude extract (1 mg/mL) was subjected to C-18 reversed-phase column chromatography on Thermo Scientific DionexUltiMate 3000 UHPLC (Thermo Fisher Scientific, Waltham, MA, USA) equipped with a diode array detector (DAD) and high-resolution mass spectrometry with electrospray ionization source (ESI-HRMS; Impact HD Mass Spectrometer, Bruker, Billerica, MA, USA). HPLC separation was performed on reversed phase Kinetex Phenomenex C_18_ column (150 × 4.6 mm, 2.6 µm) with H_2_O/acetonitrile acidified with 0.1% HCOOH as a mobile phase. Flow rate during analysis was 0.6 mL/min. The gradient was as follows: H_2_O/acetonitrile 85/15 (0 min), 85/15 (in 1 min), 0/100 (in 20 min.), 0/100 (in 25 min.), and 85/15 (in 30 min.). The HPLC was connected to a high-resolution mass spectrometer (Bruker Impact HD) with following settings: dry temperature 200 °C; drying gas flow 12 L/min; nebulizer 3 bar; capillary voltage 4500 V; endplate offset 500 V. The spectra were collected in the range 20–2000 *m*/*z* with spectra rate 2 Hz. The CID was set as a ramp from 20 to 60 eV on masses 200–1200, respectively. Calibration was performed using LockMass 622 as internal calibration solution and CH_3_COONa at the beginning of each analysis.

### 3.5. Molecular Networking

The raw analytical data were elaborated and studied using molecular networking (Global natural product social networking, GNPS). The raw data were converted to mzXML format using MSConvert from the ProteoWizard suite (http://proteowizard.sourceforge.net/tools.shtml) [42]. The molecular network was created using the Global Natural Products Social Molecular Networking (GNPS) online workflow [43]. The data were filtered by removing all MS/MS peaks within +/− 17 Da of the precursor *m*/*z*. MS/MS spectra were window-filtered by choosing only the top six peaks in the +/− 50 Da window throughout the spectrum. The data were then clustered with MS-Cluster with a parent mass tolerance of 0.1 Da and a MS/MS fragment ion tolerance of 0.025 Da to create consensus spectra. Consensus spectra containing less than 2 spectra were discarded. A network was then created where edges were filtered to have a cosine score above 0.65 and more than four matched peaks. Edges between two nodes were kept in the network if and only if each of the nodes appeared in each other’s respective top 10 most similar nodes. The spectra in the network were then searched against GNPS’ spectral libraries. The library spectra were filtered in the same manner as the input data. All matches kept between network spectra and library spectra were required to have a score above 0.7 and at least four matched peaks. Analogue search was enabled against the library with a maximum mass shift of 200 Da.

### 3.6. Isolation of Compound **2a** and **2b** from Brasilonema CT11

In order to obtain larger biomass volume, the strain was cultivated in 100 L cultivation unit twice to obtain 30 g of freeze-dried biomass. Crude extract was prepared as described previously in Section 3.2 to obtain 5 g of crude extract. The obtained crude extracts were fractionated using reversed-phase flash column chromatography, eluting with a mixture of H_2_O/CH_3_CN (from 0 to 100%) and then with 100% of MeOH, to afford twenty fractions (Fr1–Fr20) [44]. Fraction 15 eluting with 80% of CH_3_CN was found to be enriched with APT compounds and hence, was further fractionated using Sephadex LH20 to obtain ten fractions using CH_3_Cl_2_/MeOH (1:1). Fraction 3 was further purified using semi-preparative reversed-phase column chromatography, eluting with 60% of CH_3_CN, to obtain compounds **2a** and **2b**. 

Compound (**2a**) HRESIMS: tR = 20 min; [M + H]^+^ *m*/*z* 803.4520, calcd. 803.4411 (Figure S3); ^1^H NMR (700 MHz, CD3OD): δ 9.21 (1H, d, *J* = 8.8), δ 8.97 (1H, d, *J* = 3.2), δ 8.40 (2H, br s), δ 7.90 (1H, s), δ 7.82 (1H, d, *J* = 8.0), δ 7.78 (1H, br s), δ 7.57 (1H, d, *J* = 7.9), δ 7.31 (1H, d, *J* = 8.1), δ 7.21 (2H, t, *J* = 7.6), δ 7.14 (1H, t, 7.6), δ 7.11 (2H, d, *J* = 7.6), δ 7.09 (1H, br d, *J* = 7.6), δ 7.05 (1H, s), δ 7.02 (1H, t, *J* = 7.4), δ 5.05 (1H, dt, *J* = 11.2, 5.0, 3.7), δ 4.60 (1H, q, *J* = 6.7), δ 4.50 (1H, ddd, *J* = 12.5, 8.7, 2.9), δ 4.18 (1H, br s), δ 4.15 (1H, t, *J* = 4.6), δ 4.04 (1H, dd, *J* = 9.6, 4.9), δ 3.75 (1H, m), δ 3.38 (1H, dd, *J* = 13.7, 3.1), δ 3.27 (2H, m), δ 2.92 (1H, br d, *J* = 12.6), δ 2.86 (1H, t, *J* = 13.2), δ 2.25 (1H, m), δ 1.96 (1H, m), δ 1.93 (1H, m), δ 1.75 (1H, m), δ 1.57 (3H, s), δ 1.51 (2H, m), δ 1.45 (1H, m), δ 1.38 (2H, m), δ 1.26 (1H, m), δ 1.17 (3H, d, *J* = 6.8), δ 0.98 (3H, d, *J* = 6.9), δ 0.93 (3H, t, *J* = 7.4), δ 0.26 (3H, d, *J* = 6.7). 

Compound (**2b**): HRESIMS: tR = 20 min; [M + H]^+^ *m*/*z* 803.4510, calcd. 803.4411 (Figure S4); ^1^H NMR (700 MHz, CD_3_OD): δ 10.4 (1H, s, NH-Trp), δ 9.2 (1H, d, *J* = 8.7, Phe-NH), δ 9.0 (1H, d, *J* = 3.4, NH-Trp), δ 8.2 (1H, br s, NH-Val, NH-CO-2H), δ 7.9 (1H, d, *J* = 8.6, NH-Lys), δ 7.7 (1H, br s, NH-Leu), δ 7.6 (1H, d, *J* = 8.1), δ 7.3 (1H, d, *J* = 8.1), δ 7.2 (2H, t, *J*= 7.4), δ 7.14 (1H, t, *J* = 7.4), δ 7.09 (t, 3H, overlapped), δ 7.04 (1H, s), δ 7.0 (1H, t, *J*= 7.4), δ 5.1 (1H, m, CH-α, Trp), δ 4.6 (1H, q, *J* = 6.7, CH-α, NMeAla), δ 4.47 (1H, ddd, *J* = 3.1, 8.5, 12.1, CH-α Phe), δ 4.3 (1H, m, CH-α, Leu), δ 4.2 (1H, d, *J* = 3.8), δ 4.16 (1H, t, *J* = 4.9, CH-α, Lys), δ 3.8 (1H, m), δ 3.4 (1H, dd, *J* = 3.1, 13.5), δ 3.3 (1H, d, *J*= 5.1), δ 2.9 (1H, br d), δ 2.85 (1H, t, *J*= 13.3), δ 2.25 (1H, m), δ 1.95 (1H, tt, *J* = 3.4, 13.8), δ 1.87 (1H, m), δ 1.76 (2H, overlapped), δ 1.73 (1H, overlapped), δ 1.62 (1H, m), δ 1.58 (3H, s, N-CH_3_), δ 1.55 (1H, m), δ 1.47 (1H, m), δ 1.27 (1H, m), δ 1.05 (3H, d, *J* = 6.4), δ 1.0 (6H, dd, *J* = 1.9, 6.6), δ 0.92 (3H, d, *J* = 6.8), δ 0.25 (3H, d, *J* = 6.6).

### 3.7. Antiproliferative Activity 

The human cervical cancer line HeLa was obtained from Mgr. David Sedlák, Ph.D. (Institute of Molecular Genetics of ASCR, v.v.i. Czech Republic) and were maintained at 37 °C in a humidified incubator with 5% CO_2_. Cells were cultured in DMEM cultivation medium (Gibco Life Technologies) supplemented with 10% FBS (Gibco Life Technologies), 1% antibiotics (Gibco Life Technologies), and l-glutamine 2 mM (Gibco Life Technologies). The HeLa cells were plated (transparent 96-well cell culture plate, flat bottom) at a concentration of 1 × 10^4^ cells per well one day prior to cytotoxicity experiment. The exposure solutions were prepared to obtain final concentration of the compounds 20, 10, 5, 2.5, 1.25, and 0.625 µM per well. Two technical replicates in one experiment were performed and final concentration of DMSO in the tested wells did not exceed 0.5%. Staurosporine (Sigma, S5921, St. Louis, MO, USA) was used as positive control at 1 µM concentration. After 48 h of incubation MTT assay [45] was performed as the endpoint measurement. Three independent biological experiments were performed for each compound and finally the viability index was expressed as a ratio between the absorbance values of the treated and control cells in percent. 

### 3.8. Data Deposition

The strain *Brasilonema* sp. CT11 has been deposited to culture collection of autotrophic organisms (CCALA) under the strain number CCALA 1130. The genomic assembly is available under NCBI accession number JABXYX010000000, and the anabaenopeptin biosynthetic gene cluster is available under accession number MT670293. The mass spectrometry data was deposited on MassIVE public repository (MSV000085797). The molecular networking job can be publicly accessed at https://gnps.ucsd.edu/ProteoSAFe/status.jsp?task=537f43651a084553ad560eac52895539.

## Figures and Tables

**Table 1:** Product ion spectra data for compounds **1** (*m*/*z* 789.4425 [M + H]^+^), **2a/2b** (*m/z* 803.4417 [M + H]^+^), and **3** (*m*/*z* 817.4425 [M + H]^+^).

Product Ion Assignment	1 (*m*/*z*)	Error, ppm	2a/2b (*m*/*z*)	Error, ppm	3 (*m*/*z*)	Error, ppm
Lys fragment	84.0810	2.6	84.0808	0.7	84.0808	0.2
MeAla + CO + H^+^	114.0549	0.1	114.0551	1.1	114.0550	0.0
Trp fragment	130.0650	0.7	130.0653	1.0	130.0651	0.1
Trp-MeAla + H^+^	272.196	0.6	272.1399	2.1	272.1394	0.6
Trp-MeAla-Val + H^+^	371.2087	2.4	-		-	
Trp-MeAla-Ile/Leu + H^+^	-		385.2249	3.9	385.2234	0.0
CO-Lys-Phe-MeAla + H^+^	405.2141	2.1	405.2143	2.5	405.2138	1.4
Val-CO-Lys-Phe-MeAla + H ^+^	504.2811	1.0	504.2830	2.7		
Ile/Leu-CO-Lys-Phe-MeAla + H ^+^					518.2973	3.0
Val-CO-Lys-(Val)-(Phe-MeAla) + H^+^	603.3497	0.6	-		-	
Val-CO-Lys-(Ile/Leu)-(Phe-MeAla) + H^+^	-		617.3677	4.0	-	
Ile/Leu-CO-Lys-(Ile/Leu)-(Phe-MeAla) + H^+^	-		-		631.3797	2.7
Val-CO-[Lys-Val-Trp-MeAla-Phe] + H^+^	789.4304	1.3	-		-	
Val-CO-[Lys-Ile/Leu-Trp-MeAla-Phe] + H^+^	-		803.4469	2.3	-	
Ile/Leu-CO-[Lys-Ile/Leu-Trp-MeAla-Phe] + H^+^	-		-		817.4612	0.6

## Supplementary Materials

The following are available online.
