# Supplementary material for: Discovery of Unusual Cyanobacterial Tryptophan-Containing Anabaenopeptins by MS/MS-Based Molecular Networking
Source: Molecules. 2020 Aug 20;25(17):3786. doi: 10.3390/molecules25173786 (PMC7503407; doi:10.3390/molecules25173786)
Supplement: Supplementary file 1 [file molecules-25-03786-s001.pdf]

Supplementary Materials

Discovery of Unusual Cyanobacterial Tryptophan-Containing Anabaenopeptins by MS/MS-Based Molecular Networking

Subhasish Saha [1,†], Germana Esposito [2,†], Petra Urajová [1], Jan Mareš [1,3], Daniela Ewe [1], Alessia Caso [2], Markéta Macho [1,4], Kateřina Delawská [1,4], Andreja Kust [1,3], Pavel Hrouzek [1], Josef Juráň [4,5], Valeria Costantino [2,*] and Kumar Saurav [1,*]

1 Laboratory of Algal Biotechnology-Centre Algatech, Institute of Microbiology of the Czech Academy of Sciences, 37981, Třeboň, Czech Republic; saha@alga.cz (S.S.); urajova@alga.cz (P.U.); mares@alga.cz (J.M.); ewe@alga.cz (D.E.); macho@alga.cz (M.M.); delawska@alga.cz (K.D.); kust@alga.cz (A.K.); hrouzek@alga.cz (P.H.)

2 Task Force Big Fed2, The Blue Chemistry lab, Università degli Studi di Napoli, 80131, Napoli, Italy; germana.esposito@unina.it (G.E.); alessia.caso@unina.it (A.C.)

3 Institute of Hydrobiology, Biology Centre of the Czech Academy of Sciences, 37005, České Budějovice, Czech Republic

4 Faculty of Science, University of South Bohemia in České Budějovice, Branišovská 1760, 37005, České Budějovice, Czech Republic. josef.juran@ibot.cas.cz

5 Institute of Botany of the Czech Academy of Sciences, 252 43, Průhonice, Czech Republic

† These authors contributed equally to this work

* Correspondence: valeria.costantino@unina.it (V.C.), saurav@alga.cz (K.S.); Tel.: +420-737-003-359 (K.S.).

Academic Editor: Derek J. McPhee

Received: 21 July 2020; Accepted: 19 August 2020; Published: 20 August 2020

Table S1. Biosynthetic gene clusters (BGCs) predicted from the genome of *Brasilonema* sp. CT11 using antiSMASH. Out of the 36 clusters identified, four clusters possess 100% similarity with other known BGCs whereas only two clusters were detected with more than 75% similarity with BGCs of known compounds.

Table S2. Summary of deduced proteins in the anabaenopeptin biosynthetic pathway in *Brasilonema* sp. CT11 and their closest homologues.

Table S3. Summary of adenylation domains and their substrates involved in anabaenopeptin biosynthesis in *Brasilonema* sp. CT11.

Figure S1. Microphotograph of *Brasilonema* sp. CT11 with heterocytous false-branching filaments. Scale bar represents 100 μM.

Figure S2. Molecular networking analyses of the crude extract of *Brasilonema* CT11 analyzed via LC-HRMS/MS. Labelled box represents the cluster containing APT molecules.

Figure S3. HR-MS/MS product ion spectra of protonated anabenopeptin 802 **2a** and **2b** from *Brasilonema* CT11.

Table S4. NMR data of anabenopeptin 802a (**2a**) (700 MHz, CD_3OD).

Figure S4. ^{1}H-NMR spectrum of anabenopeptin 802a (**2a**) (700 MHz, CD_3OD).

Figure S5. COSY spectrum of anabenopeptin 802a (**2a**) (700 MHz, CD_3OD).

Figure S6. NOESY spectrum of anabenopeptin 802a (**2a**) (700 MHz, CD_3OD).

Figure S7. HSQC spectrum of anabenopeptin 802a (**2a**) (700 MHz, CD_3OD).

Figure S8. HMBC spectrum of anabenopeptin 802a (**2a**) (700 MHz, CD_3OD).

Figure S9. TOCSY spectrum of anabenopeptin 802a (**2a**) (700 MHz, CD_3OD).

Table S5. NMR data of anabenopeptin 802b (**2b**) (700 MHz, CD_3OD).

Figure S10. ^{1}H-NMR spectrum of anabenopeptin 802b (**2b**) (700 MHz, CD_3OD).

Figure S11. COSY spectrum of anabenopeptin 802b (**2b**) (700 MHz, CD_3OD).

Figure S12. NOESY spectrum of anabenopeptin 802b (**2b**) (700 MHz, CD_3OD).

Figure S13. HSQC spectrum of anabenopeptin 802b (**2b**) (700 MHz, CD_3OD).

Figure S14. HMBC spectrum of anabenopeptin 802b (**2b**) (700 MHz, CD_3OD).

Figure S15. TOCSY spectrum of anabenopeptin 802b (**2b**) (700 MHz, CD_3OD).

Table 1. BGCs predicted from this strain using antiSMASH. Out of the 36 clusters identified, four clusters possess 100% similarity with other known BGCs, whereas only two clusters were detected with more than 75% similarity with BGCs of known compounds.

**Metabolite Class**	***Brasilonema* CT11**	**Most similar known cluster (%)**
PKS I	01	Merocyclophane C/D (22%)
NRPS	12	Anabaenopeptin 908/915 (100%)
PKS-NRPS Hybrid	06	Nostopeptolide A2 (100%)
Terpene	03	Geosmin (100%)
RiPP	02	-
Bacteriocin	04	-
Indole	01	Staurosporine (26%)
Mixed	04	-
Others	03	-

Table 2. Summary of deduced proteins in the anabaenopeptin biosynthetic pathway in *Brasilonema* sp. CT11 and their closest homologues.

Anabaenopeptin Pathway			Closest Homologue (BLASTp)				Top BLASTp Hit with Known Function			
**Protein**	**Size (aa)**	**Proposed Function**	**Accession**	**Organism**	**Identity (%)**	**Function**	**Accession**	**Organism**	**Identity (%)**	**Function**
AptA	2201	NRPS	WP_171976800.1	*Brasilonema* (multispecies)	89	NRPS (hypothetical)	AVK43380.1	*Nostoc* sp. N135.9.1	81	NRPS—AptA (anabaenopeptin pathway)
AptB	1069	NRPS	WP_073634533.1	*Scytonema* sp. HK-05	87	NRPS (hypothetical)	ASR75186.1	*Nostoc* sp. KVJ2	83	NRPS—AptB (anabaenopeptin pathway)
AptC	2588	NRPS	WP_073634532.1	*Scytonema* sp. HK-05	84	NRPS (hypothetical)	AVK43394.1	*Nostoc* sp. XHIID C2	82	NRPS—AptC (anabaenopeptin pathway)
ORF1	206	unknown	WP_073634531.1	*Scytonema* sp. HK-05	88	Uma2 family endonuclease (hypothetical)			no hit	
AptD	1397	NRPS	WP_171976805.1	*Brasilonema* (multispecies)	91	NRPS (hypothetical)	AVK43292.1	*Nodularia spumigena* AV2	83	NRPS—AptD (anabaenopeptin pathway)
AptE	736	ABC transporter	WP_171976806.1	*Brasilonema* (multispecies)	82	ATP-binding cassette domain-containing protein (hypothetical)	AVV48476.1	*Anabaena* sp. SYKE748A	71	ABC transporter—AptE (anabaenopeptin pathway)

Table 3. Summary of adenylation domains and their substrates involved in anabaenopeptin biosynthesis in *Brasilonema* sp. CT11.

Protein	Domain (aa range)	Closest Annotated BLAST Hit (aa range)	Identity (%)	Predicted Substrate (antiSMASH)	Incorporated aa Residue in Anabaenopeptin
AptA	A0 (46–449)	AptA1, *Nostoc* sp. 268, AVK43337.1 (47–446)	83	Val/Ile	L-Val/L-Leu
	A1 (1130–1537)	AptA2, *Nostoc* sp. N135.9.1, AVK43380.1 (1121–1523)	82	Lys	D-Lys
AptB	A2 (450–848)	AptB, *Nostoc* sp. XHIID C2, AVK43393.1 (470–867)	84	Leu/Ile	L-Leu/L-Ile/L-Val
AptC	A3 (511–905)	AptD, *Nostoc* sp. KVJ2, ASR75188.1 (544–937)	80	Leu	L-Trp
	A4 (1563–1972)	AptC, *Nostoc* sp. XHIID C2, AVK43394.1 (1569–1967)	82	Ala	L-*N*MeAla
AptD	A5 (545–939)	AptD, *Nodularia spumigena* 309, AVK43275.1 (546–937)	82	Phe	L-Phe

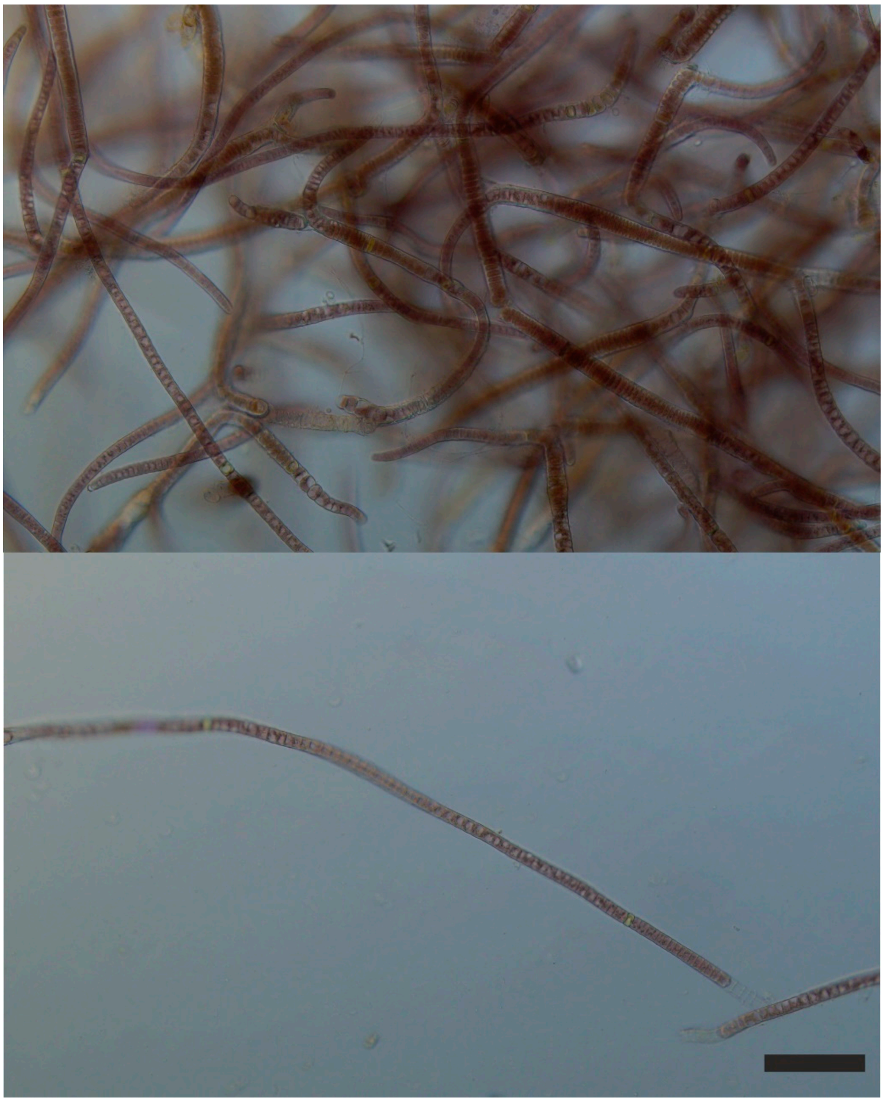

Figure S1. Microphotograph of *Brasilonema* sp. CT11 with heterocytous false-branching filaments. Scale bar represents 100 μM.

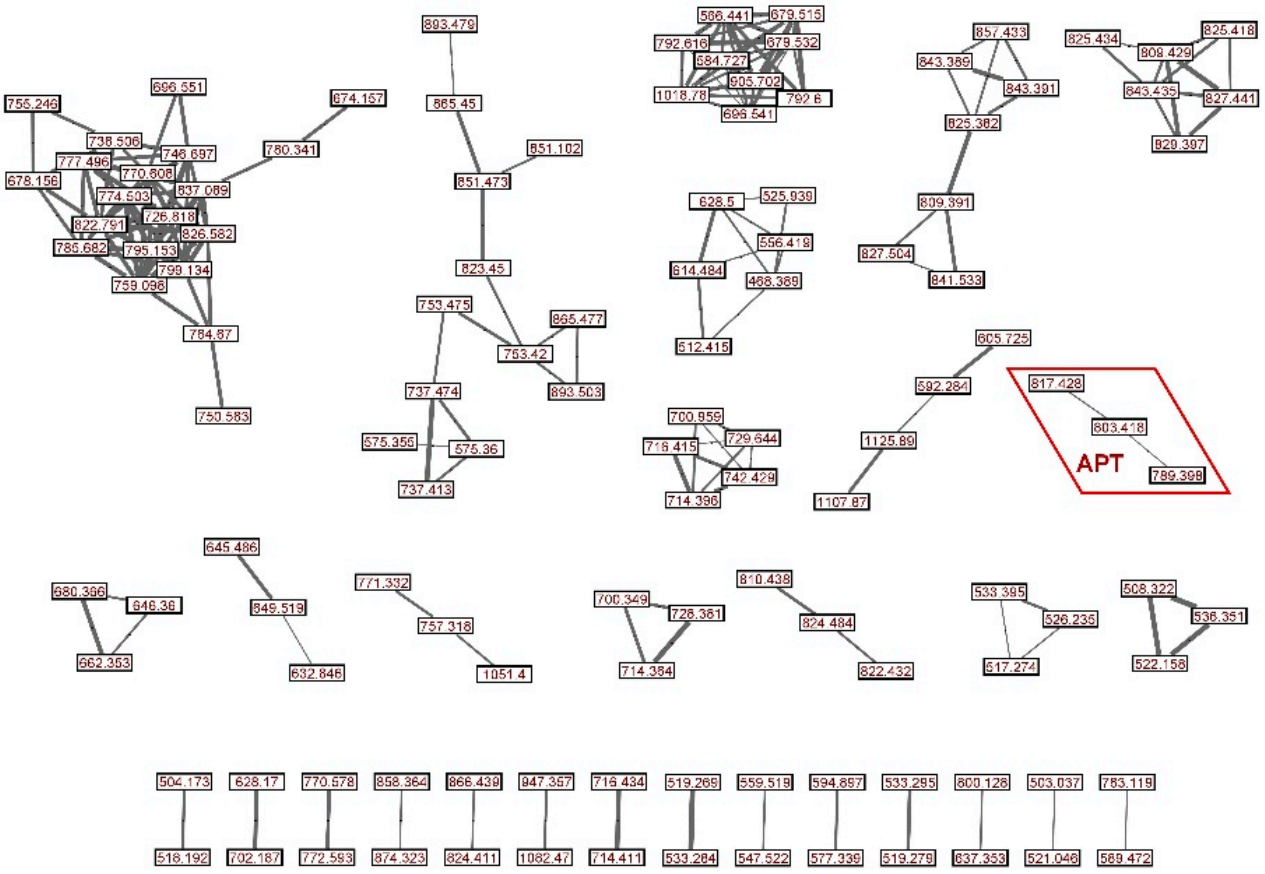

Figure S2. Molecular networking analyses of the crude extract of *Brasilonema* CT11 analyzed via LC-HRMS/MS. Labelled box represents the cluster containing APT molecules.

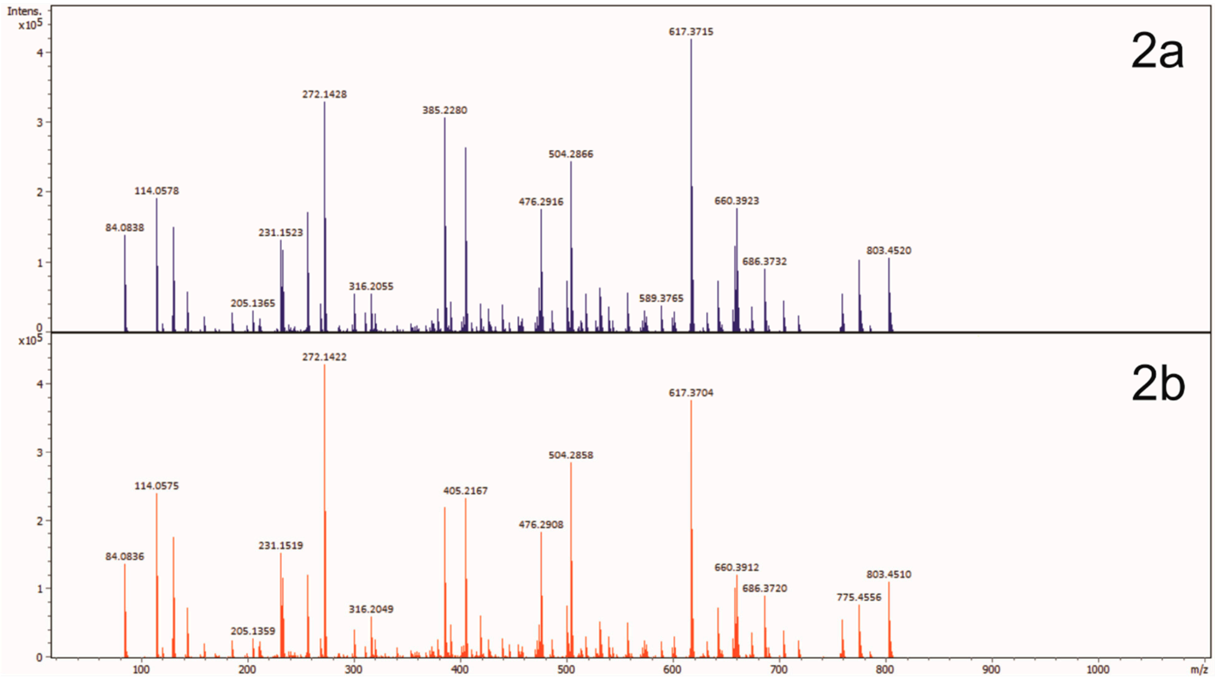

Figure S3. HR-MS/MS product ion spectra of protonated anabenopeptin 802 **2a** and **2b** from *Brasilonema* CT11.

Table 4. NMR data of anabenopeptin 802a (**2a**) (700 MHz, CD_3OD).

	Position	d_C, type		d_H, Mult (J in Hz)	HMBC [a]	NOESY
Val	NH			8.40, br s		
	1	175.9, C		-		
	2	59.7, CH		4.18, br s		
	3	31.6, CH		2.25, m		4, 5
	4	17.3, CH_3		0.91, d (6.9)	2, 3, 5	2, 3
	5	19.8, CH_3		0.98, d (6.9)	2, 3, 4	2, 3
C=O	6	159.6, C		-		
Lys	NH			8.40, br s		
	1	175.9, C		-		
	2	56.0, CH		4.15, t (4.6)	1, 3, 4, 6, 7	3a
	3	32.3, CH_2	a	1.75, m	1, 2	
			b	1.93, m	1	
	4	21.5, CH_2	a	1.26, m		
			b	1.45, m		
	5	35.6, CH_2		1.51, m		
	6	39.8, CH_2	a	2.92, br d (12.6)		6b
			b	3.75, m		6a
	NH			7.82, d (8.0)		
Phe	NH			9.21, d (8.8)		
	1	174.0, C		-		
	2	56.6, CH		4.5, ddd (12.5, 8.7, 2.9)	1, 3, 4	3a
	3	38.6, CH_2	a	2.86, t (13.2)	2, 4, 5/9	
			b	3.38, dd (13.7, 3.1)	4, 5/9	3a
	4	139.4, C		-		
	5/9	129.9, CH		7.11, d (7.6)	3, 6, 7	2, 3a
	6/8	129.5, CH		7.21, t (7.6)	4, 5/9	
	7	127.3, CH		7.14, t (7.6)	5/9	
NMeAla	1	173.3, C		-		

	2	56.6, CH	4.60, q (6.7)	1, 3, 4	3
	3	12.5, CH_3	-0.26, d (6.7)	1, 2	2
	4	27.9, CH_3	1.57, s	2, C1-Trp	
Trp	NH		8.97, d (3.2)		
	1	174.0, C	-		
	2	51.7, CH	5.05, dt (11.2, 5.0, 3.7)	1, 3, 4	
	3	28.8, CH_2	3.27, m	1, 2, 4, 5	
	4	109.8, C	-		
	5	124.5, CH	7.05, s	3, 4, 6, 11	10
	6	128.7, C	-		
	7	112.3, CH	7.31, d (8.1)	6, 8	
	8	119.9, CH	7.02, t (7.4)	6, 7	10
	9	122.3, CH	7.09, br d (7.6)	10, 11	
	10	119.0, CH	7.57, d (7.9)	4, 6, 9, 11	5, 8, 9
	11	137.8, C	-		
	NH		7.90, s		
Ile	NH		7.78, br s		
	1	173.9, C	-		
	2	59.3, CH	4.04, dd (9.6, 4.9)	1, 3	3
	3	36.9, CH	1.96, m		
	4	15.3, CH_3	1.17, d (6.8)	2, 3	
	5	19.8, CH_2	1.38, m	6	
	6	14.0, CH_3	0.93, t (7.4)	5	2

[a] Selected HMBC correlations from proton stated to the indicated carbon.

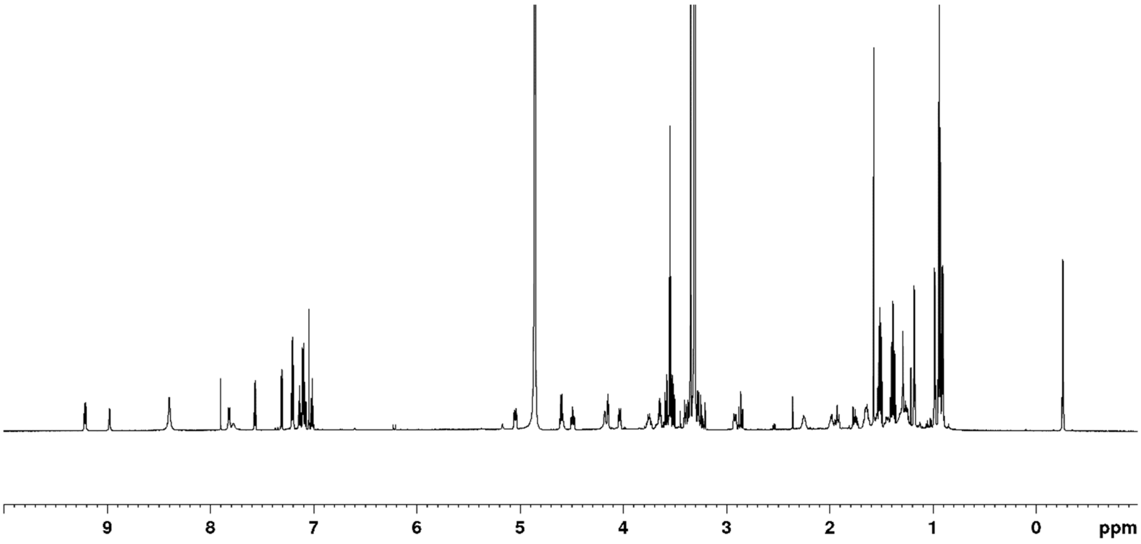

Figure S4. ^{1}H-NMR spectrum of anabenopeptin 802 (**2a**) (700 MHz, CD_3OD).

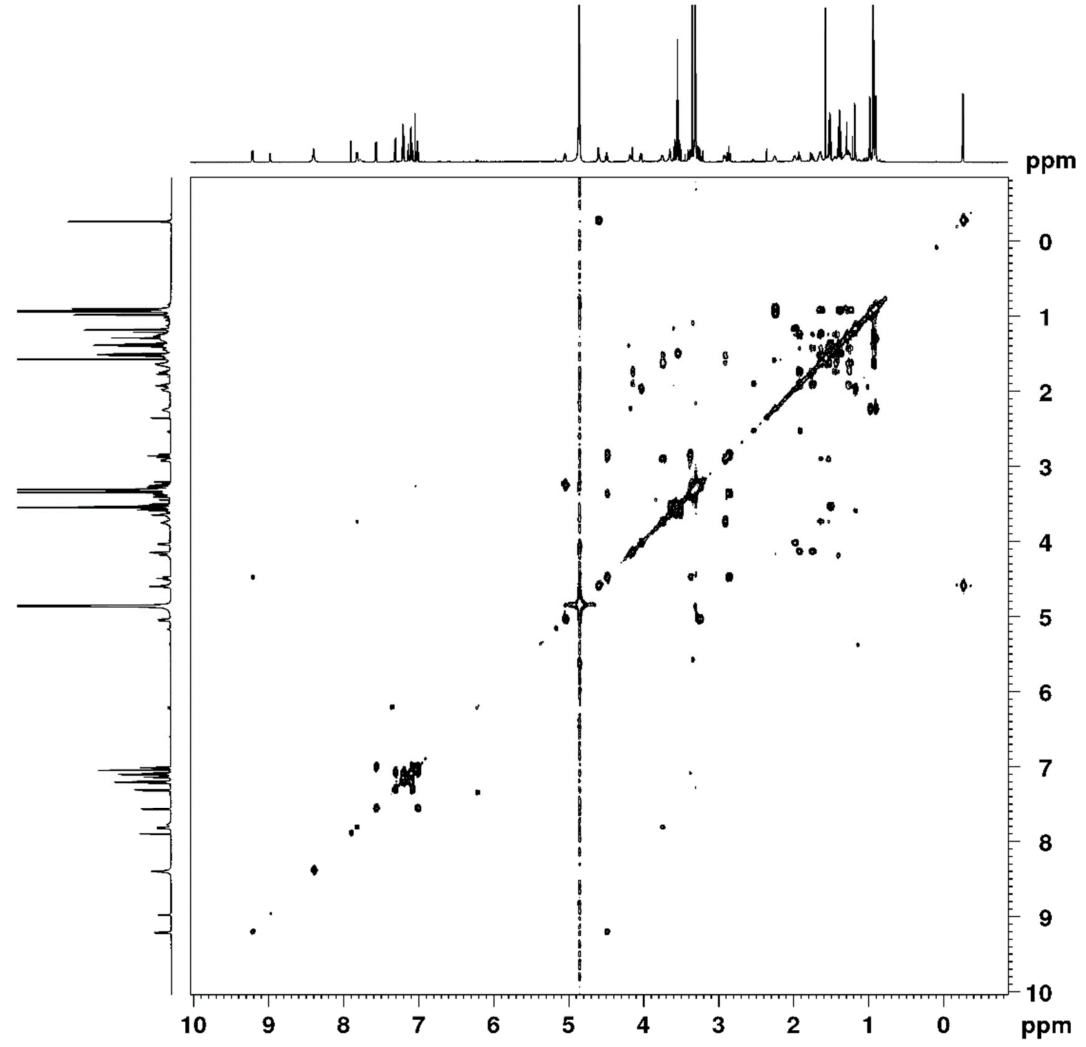

Figure S5. COSY spectrum of anabenopeptin 802 (**2a**) (700 MHz, CD_3OD).

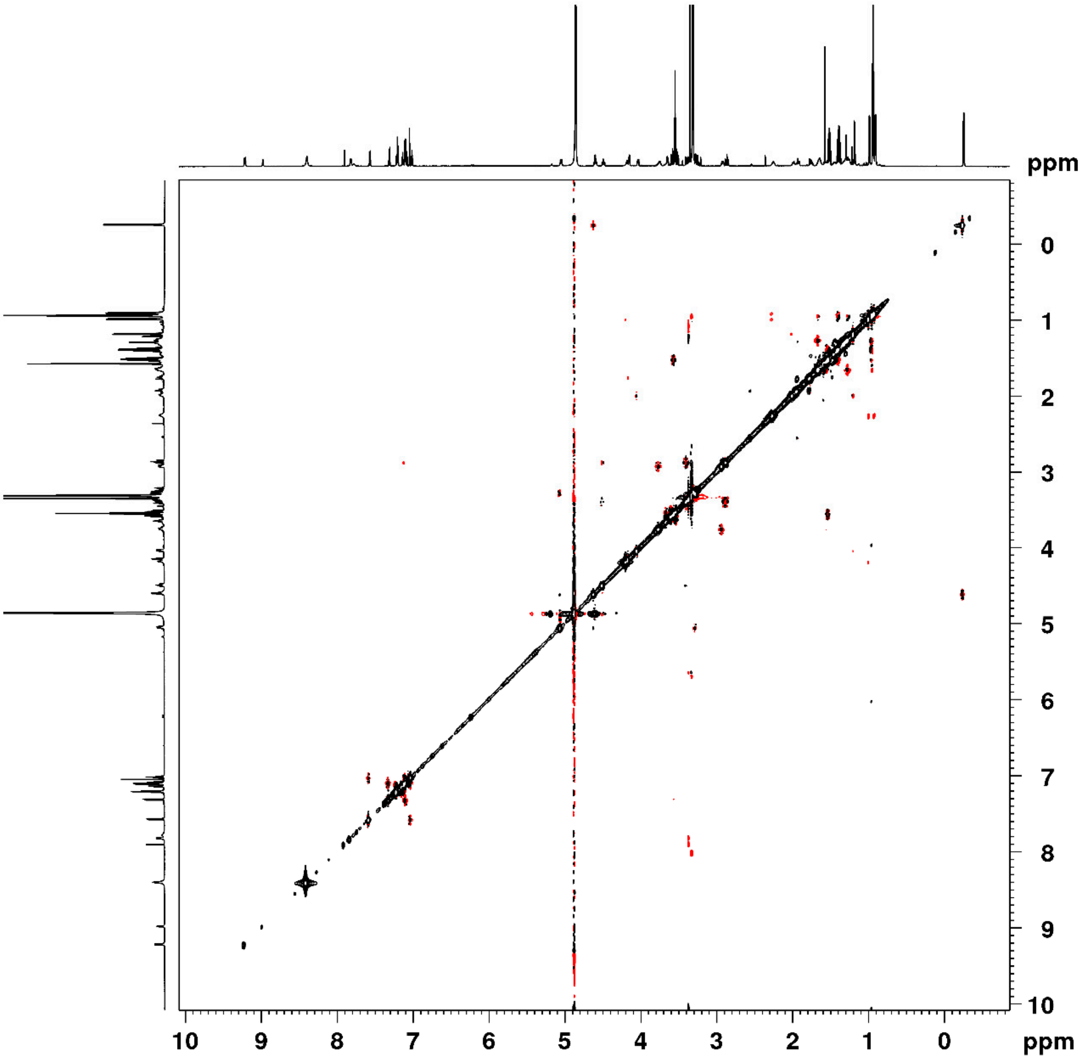

Figure S6. NOESY spectrum of anabenopeptin 802 (**2a**) (700 MHz, CD_3OD).

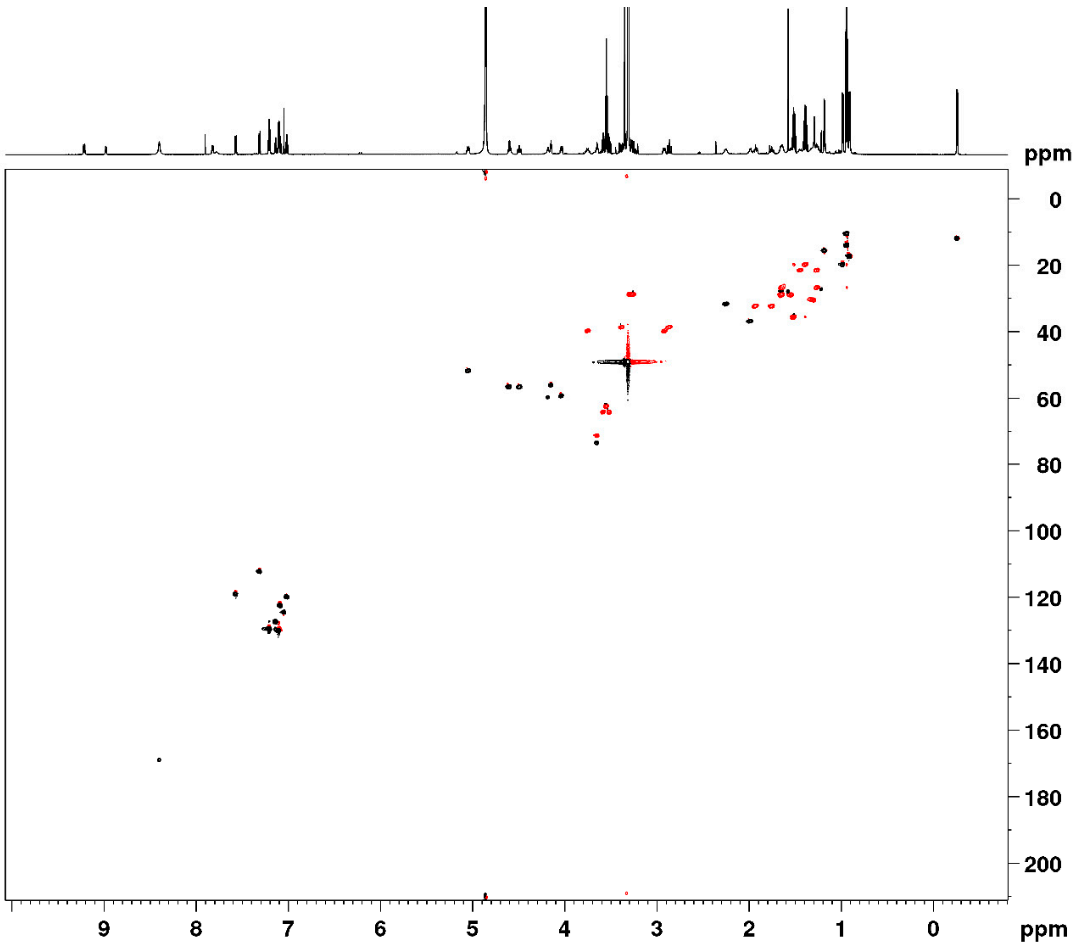

Figure S7. HSQC spectrum of anabenopeptin 802 (**2a**) (700 MHz, CD_3OD).

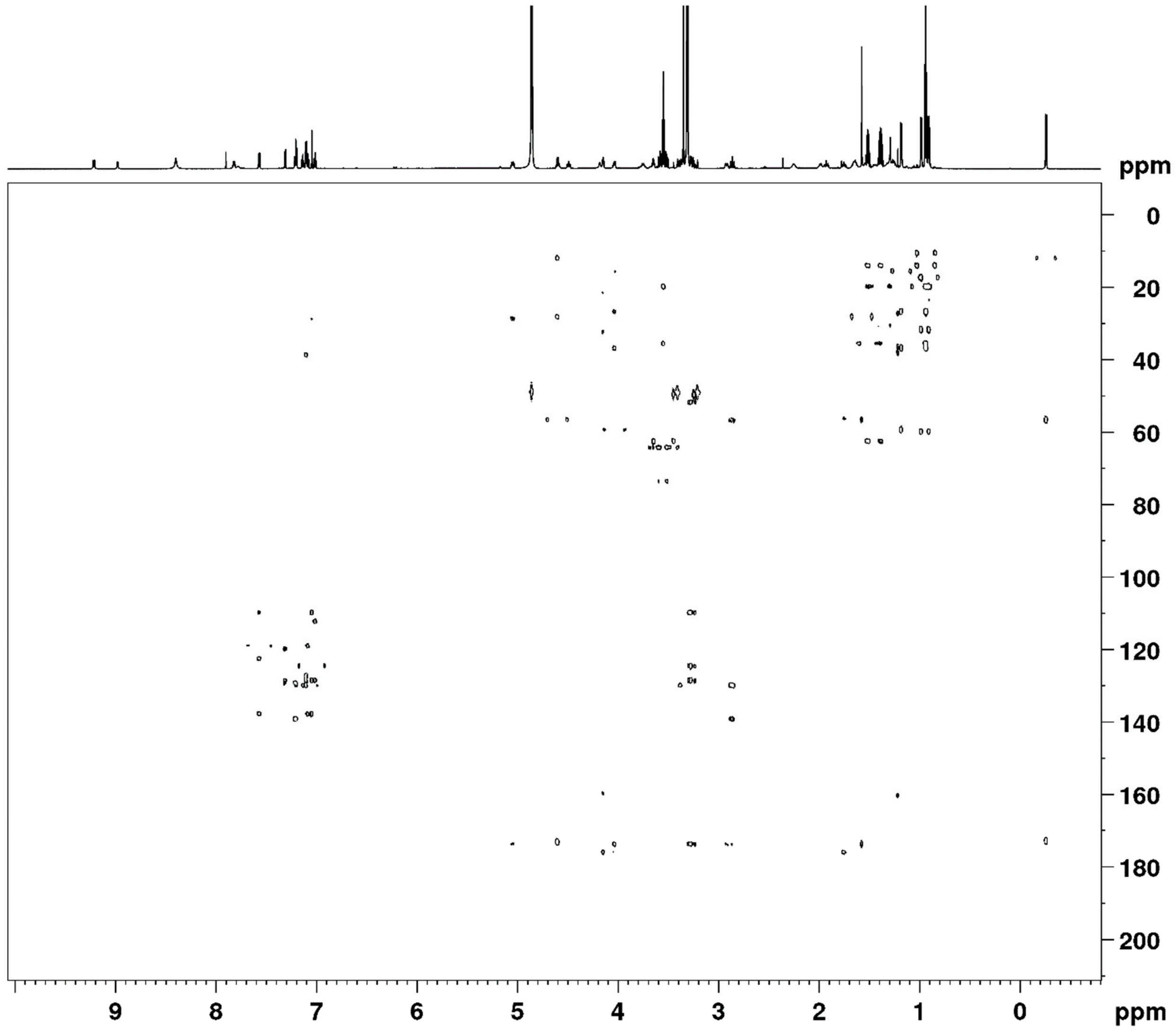

Figure S8. HMBC spectrum of anabenopeptin 802 (**2a**) (700 MHz, CD_3OD).

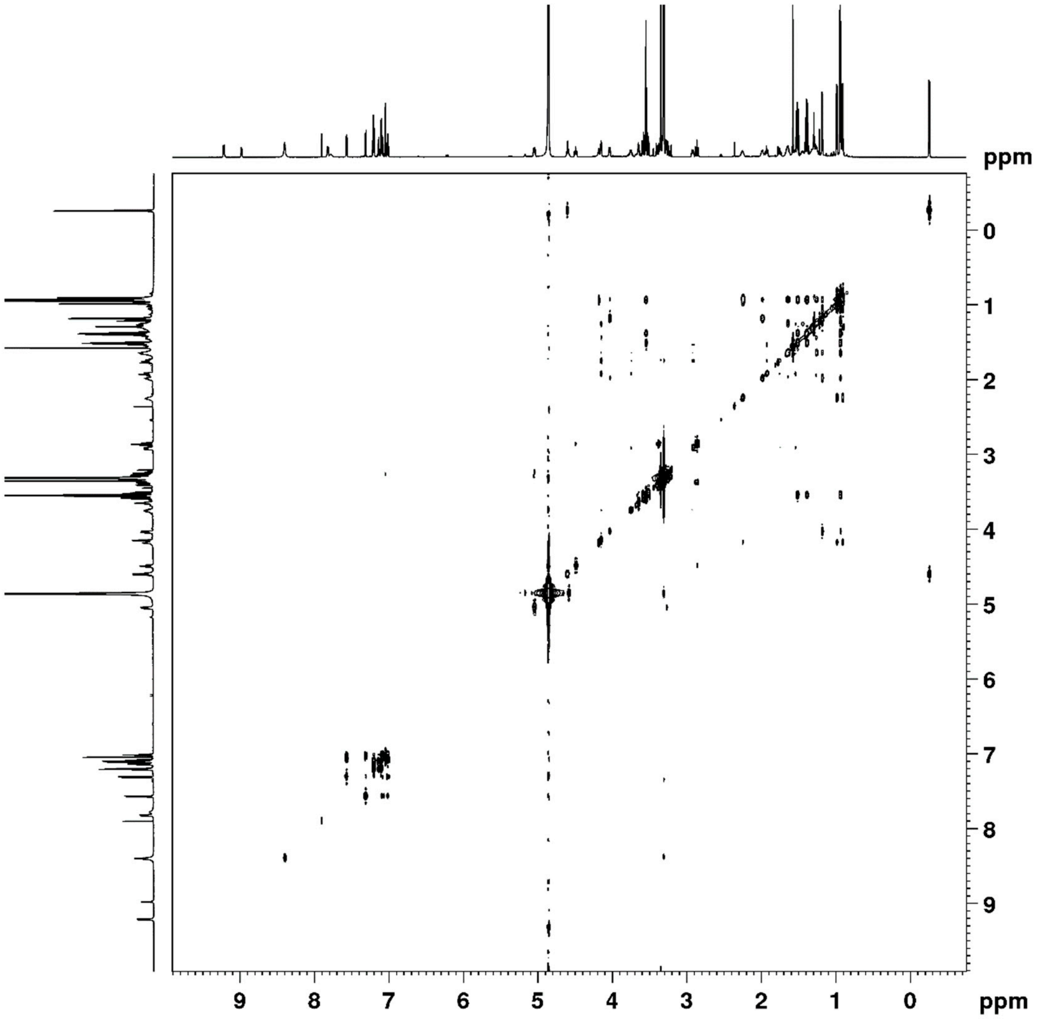

Figure S9. TOCSY spectrum of anabenopeptin 802 (**2a**) (700 MHz, CD_3OD).

Table S4. NMR data of anabenopeptin 802b (**2b**) (700 MHz, CD_3OD).

	Position	δ_C, type		δ_H, mult (J in Hz)	HMBC[a]	NOESY
Val	NH			8.20, br s		
	1	176.7, C		-		
	2	59.2, CH		4.20, d (3.8)	1, 3,7	3,5
	3	31.3, CH		2.25, m		
	4	17.2, CH_3		0.92, d (6.8)		3
	5	19.8, CH_3		1.00, dd (6.6, 1.9)		3
C=O	6	159.6, C				
Lys	NH			8.20, br s		
	1	176.3, C		-		
	2	55.9, CH		4.16, t (4.9)	1, Val-NH	3a, 4a
	3	31.1, CH_2	a	1.73, ovl		
			b	1.95, tt (13.8, 3.4)		
	4	20.2, CH_2	a	1.27, m		
			b	1.47, m		
	5	29.0, CH_2	a	1.55, m		
			b	1.62, m		
	6	39.8, CH_2	a	2.90, br d		
			b	3.80, m		
	NH			7.90, d (8.6)		5b, 6a, 6b
Phe	NH			9.20, d (8.7)		2, 3a
	1	176.3, C		-		
	2	56.9, CH		4.47, ddd (12.1, 8.5, 3.1)	1, 3a, 4	3a, 3b
	3	38.8, CH_2	a	2.85, t (13.3)		
			b	3.40, dd (13.5,3.1)		
	4	139.1, C		-		
	5/9	129.5, CH		7.20, t (7.4)		
	6/8	130.1, CH		7.09, ovl	4	2, 3a, H4-Leu, H5-Leu

	7	127.6, CH	7.14, t (7.4)		
NMeAla	1	173.5, C	-		
	2	56.5, CH	4.60, q (6.7)	3	3
	3	10.7, CH_3	-0.25, d (6.6)		
	4	27.6, CH_3	1.58, s	2	
Trp	NH		9.00, d (3.4)		
	1	173.6, C	-		
	2	51.7, CH	5.10, m		
	3	28.3, CH2	3.30, d (5.1)	1, 4, 5	2
	4	109.5, C	-		
	5	124.7, CH	7.04, s	4	
	6	128.5, C	-		
	7	119.1, CH	7.60, d (8.1)	9, 11	2, 3, H2-Leu
	8	120.1, CH	7.00, t (7.4)	6, 10	
	9	122.5, CH	7.09, ovl		
	10	112.5, CH	7.30, d (8.1)		
	11	137.8, C	-		
	NH		10.40, s		
Leu	NH		7.70, br s		
	1	174.7, C	-		
	2	53.5, CH	4.30, m	1, 3, 4	
	3	41.1, CH_2	1.76, ovl		
	4	25.4, CH	1.87, m	2, 3	
	5	22.3, CH_3	1.00, dd (6.6, 1.9)		1
	6	23.0, CH_3	1.05, d (6.4)		3, 4

[a] Selected HMBC correlations from proton stated to the indicated carbon.

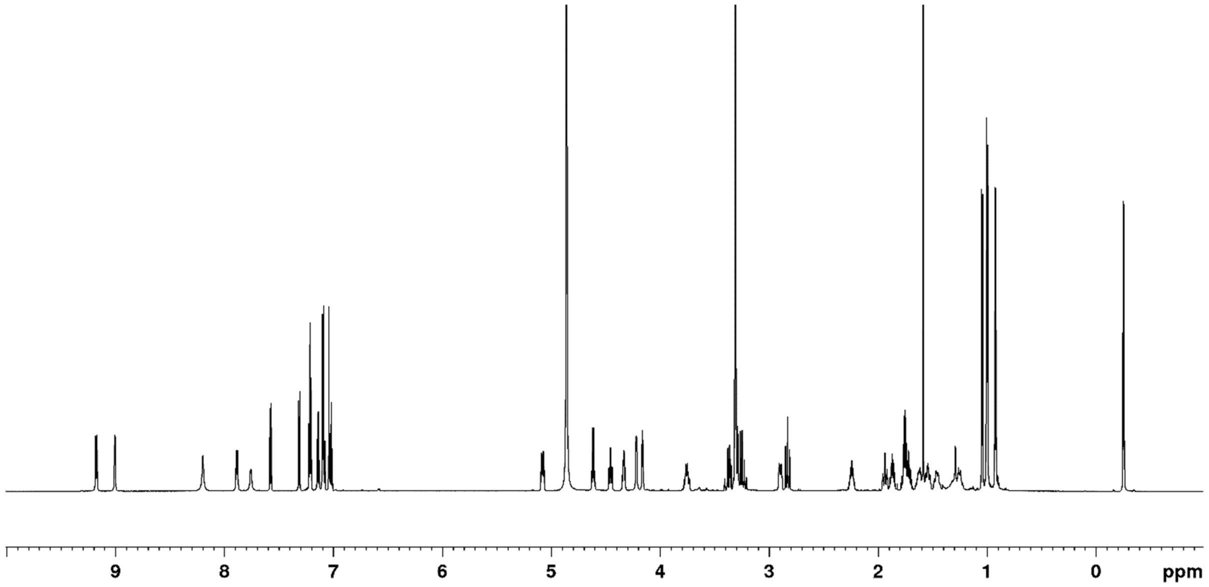

Figure S10. ^{1}H-NMR spectrum of anabenopeptin 802b (**2b**) (700 MHz, CD_3OD).

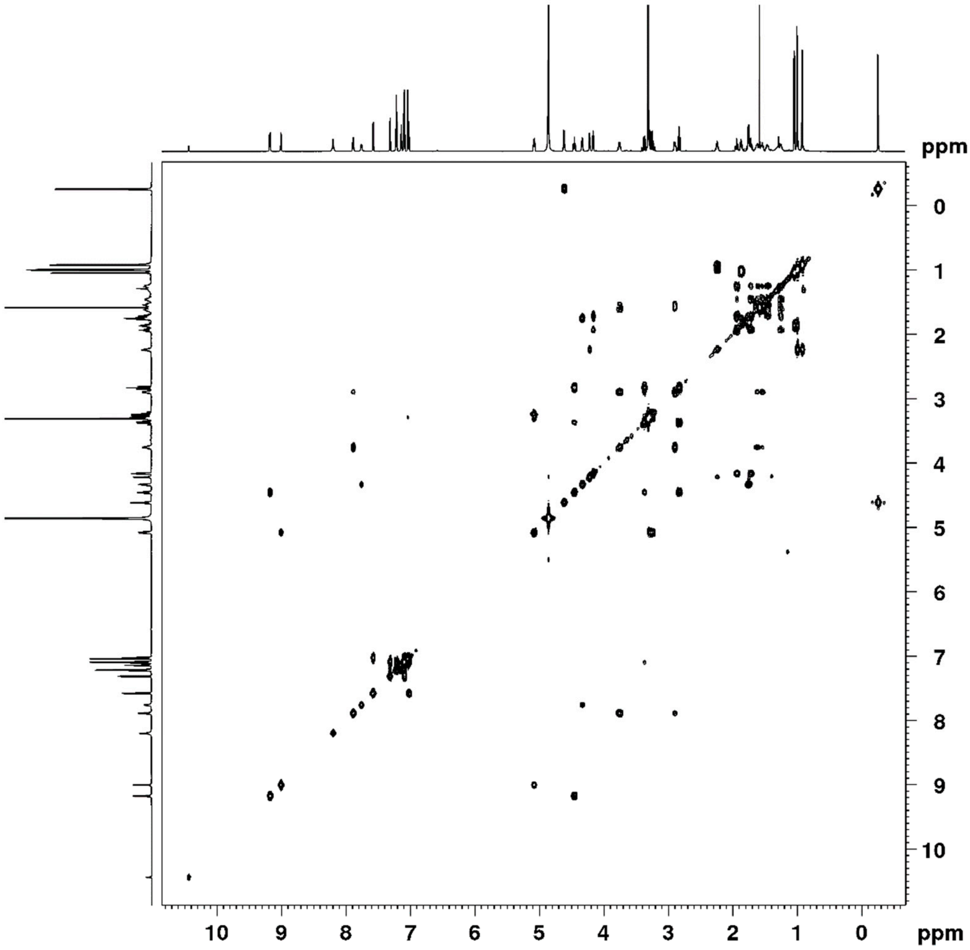

Figure S11. COSY spectrum of anabenopeptin 802b (**2b**) (700 MHz, CD_3OD).

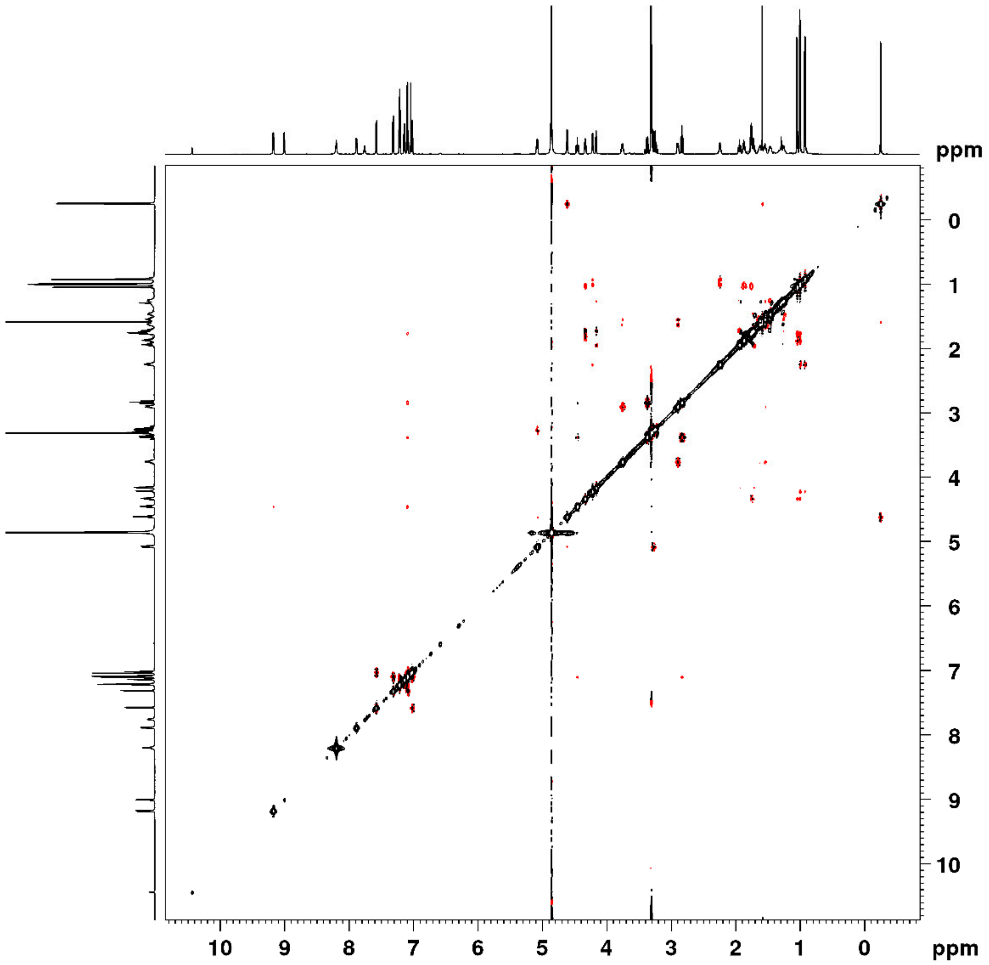

Figure S12. NOESY spectrum of anabenopeptin 802b (**2b**) (700 MHz, CD_3OD).

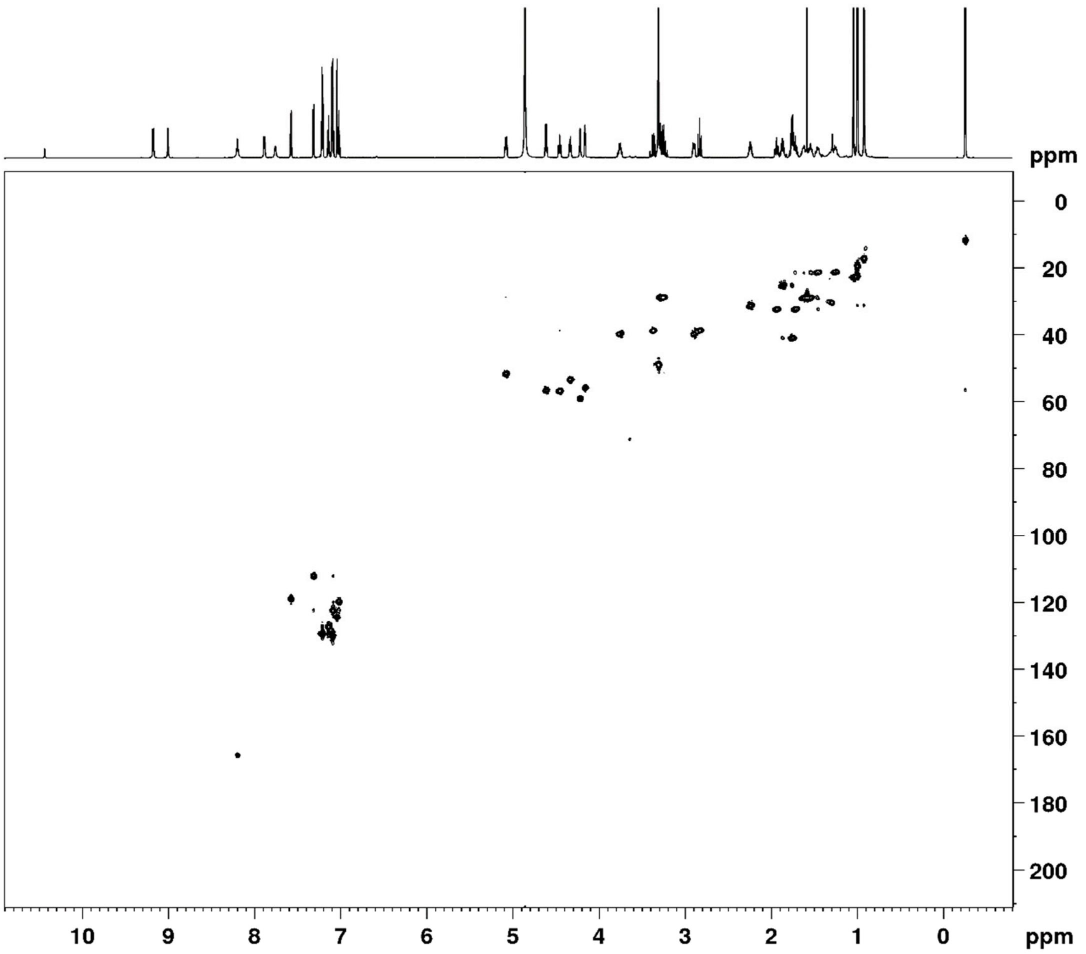

Figure S13. HSQC spectrum of anabenopeptin 802b (**2b**) (700 MHz, CD_3OD).

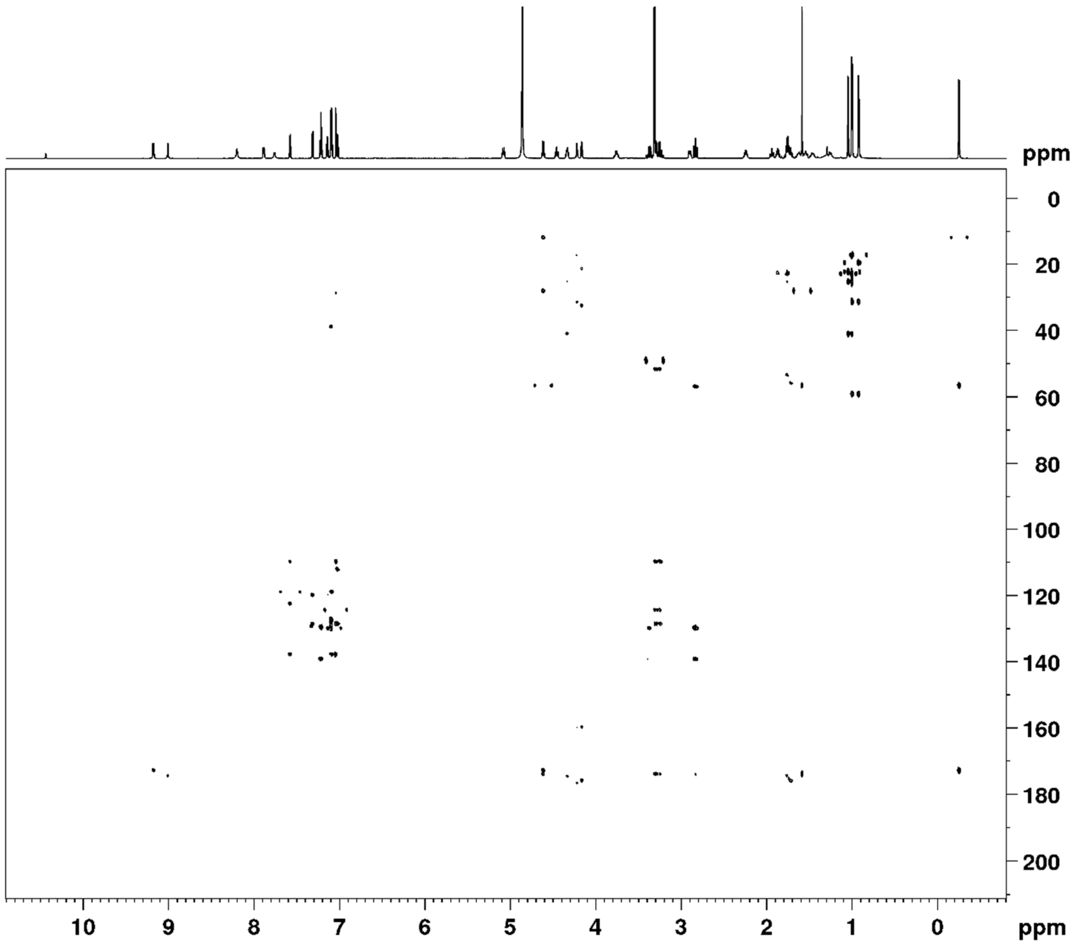

Figure S14. HMBC spectrum of anabenopeptin 802b (**2b**) (700 MHz, CD_3OD).

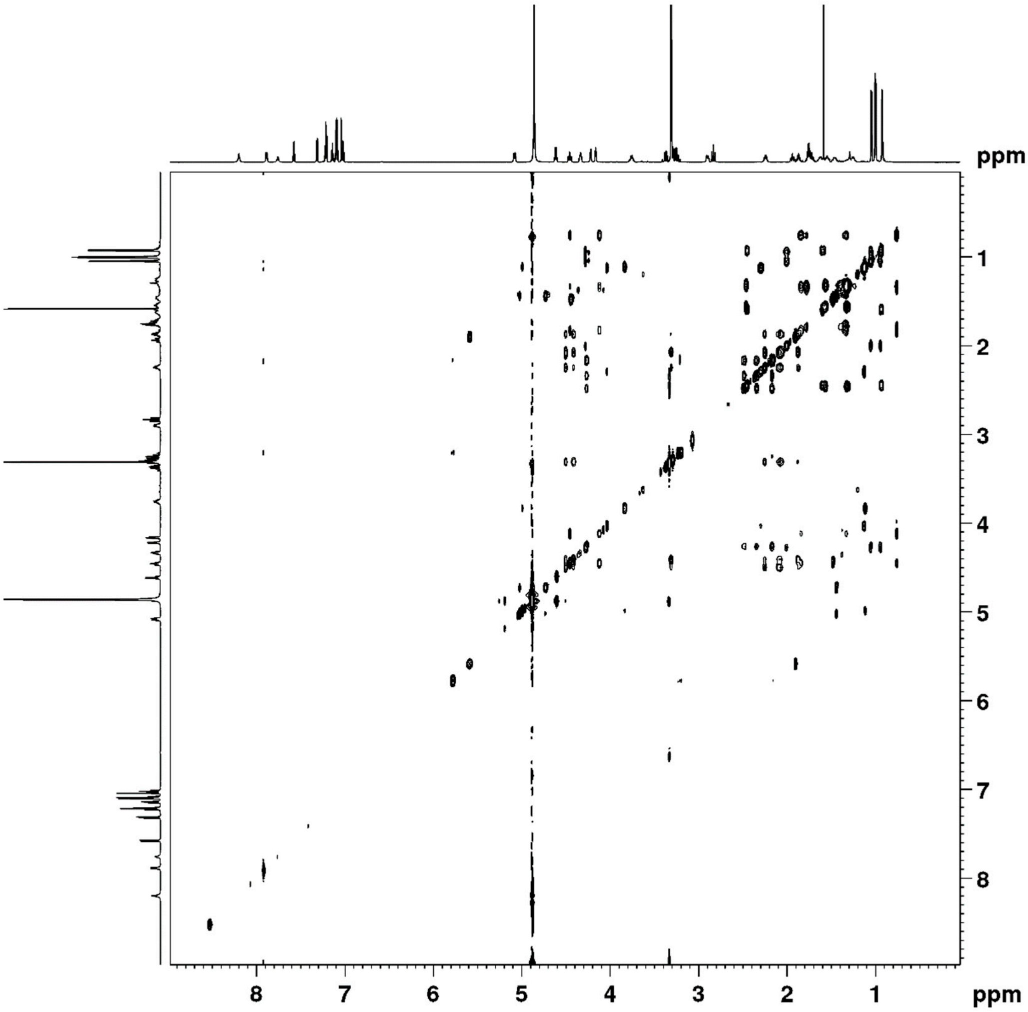

Figure S15. TOCSY spectrum of anabenopeptin 802b (**2b**) (700 MHz, CD_3OD).
